# A High-Quality Genome Assembly of *Sorghum dochna*

**DOI:** 10.3389/fgene.2022.844385

**Published:** 2022-08-12

**Authors:** Yu Chen, Yongbai Zhang, Hongjie Wang, Juan Sun, Lichao Ma, Fuhong Miao, Zixin Zhang, Yang Cheng, Jianwei Huang, Guofeng Yang, Zengyu Wang

**Affiliations:** ^1^ College of Grassland Science, Qingdao Agricultural University, Qingdao, China; ^2^ Key Laboratory of National Forestry and Grassland Administration on Grassland Resources and Ecology in the Yellow River Delta, Qingdao Agricultural University, Qingdao, China; ^3^ College of Animal Science, Qingdao Agricultural University, Qingdao, China; ^4^ Berry Genomics Corporation, Beijing, China

**Keywords:** *Sorghum dochna*, genome, assembly, comparative genome analysis, Hi-C

## Abstract

Sweet sorghum (*Sorghum dochna*) is a high-quality bio-energy crop that also serves as food for humans and animals. However, there is little information on the genomic characteristics of *S. dochna*. In this study, we presented a high-quality assembly of *S. dochna* with PacBio long reads, Illumina short reads, high-throughput chromosome capture technology (Hi-C) sequencing data, gene annotation, and a comparative genome analysis. The results showed that the genome of *S. dochna* was assembled to 777 Mb with a contig N50 of 553.47 kb and a scaffold N50 of 727.11 kb. In addition, the gene annotation predicted 37,971 genes and 39,937 transcripts in the genome of *S. dochna*. A Venn analysis revealed a set of 7,988 common gene annotations by integrating five databases. A Cafe software analysis showed that 191 gene families were significantly expanded, while 3,794 were significantly contracted in *S. dochna*. A GO enrichment analysis showed that the expanded gene families were primarily clustered in the metabolic process, DNA reconstruction, and DNA binding among others. The high-quality genome map constructed in this study provides a biological basis for the future analysis of the biological characteristics of *S. dochna*, which is crucial for its breeding.

## Introduction

*Sorghum dochna* belongs to the Gramineae family and has high sugar content in its stalks. Typically, it is a perennial crop except in frost-prone areas. Relevant historical records indicate that *S. dochna* was initially grown in India and Myanmar. During the mid-19th century, the United States introduced the *S. dochna* variety “Amber” from south China for cultivation, resulting in the annual production of *S. dochna* syrup as high as 111.56 million liters. Currently, *S. dochna* is cultivated in all continents of the world (Gnansounou et al., 2005).

Considering that most global economies are moving toward low-carbon energy sources, bio-renewable energy may replace oil and coal (Antonopoulou et al., 2008). *S. dochna* is an ideal bio-energy crop owing to its high photosynthetic efficiency, high resistance to stress, high sugar content (Erdei et al., 2009), high yield, and drought resistance. Thus, *S. dochna* can be used as an excellent silage material. In addition, it tastes delicious and is suitable for livestock consumption. Moreover, *S. dochna* as human food can be eaten raw or used as a raw material for making sugar, wine, and other related products. After threshing, *S. dochna* tassels can also be used to make brooms and cookware. Currently, *S. dochna* is economically valuable. There is a need for more insight into its biological mechanisms and genomic characteristics to more efficiently utilize its biological value.

To date, many studies have provided sufficient data for elucidating the *S. bicolor* genome (Paterson et al., 2009). In addition, the exploitation of *S. bicolor* as human food has increased worldwide. However, studies on the genomic analysis of *S. dochna* are limited. Moreover, the genomic characteristics of *S. dochna* are poorly understood. In this study, a genome assembly of *S. dochna* was constructed at the chromosome level using PacBio long-read, Illumina short-read, and high-throughput chromosome capture technology (Hi-C) sequencing data (Jin et al., 2021). This study provides valuable genomic data that can be used to conduct further research on the economic value of *S. dochna*. In addition, the findings of this study will facilitate comparative genomic analyses with other Gramineae forage plants.

## Materials and Methods

### Materials Collection

**FIGURE 1:**
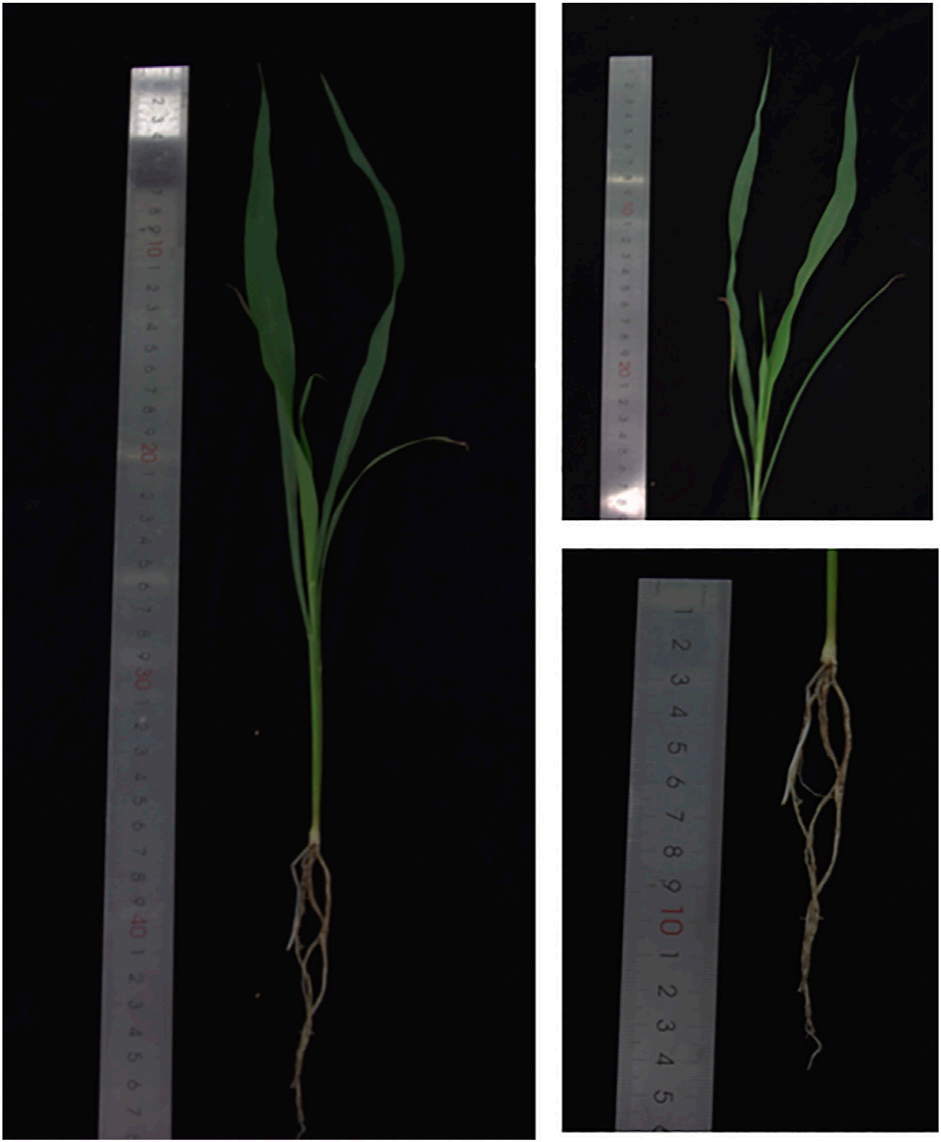
Morphological characteristics of *Sorghum dochna* as shown in photographs that display a whole plant, leaf, and root.

*S. dochna* variety De Sheng was selected and cultivated in soil at the Research Center of Grassland, Agriculture, and Animal Husbandry of Qingdao Agricultural University (Qingdao, China). The *S. dochna* seeds were washed once with distilled water and disinfected with 75% alcohol for 1 min and NaClO for 7–8 min. After that, the seeds were dried and planted in sterilized nutrient soil. The leaves of 45-day-old seedlings were harvested (Figure 1), frozen in liquid nitrogen, and then stored at −80°C for subsequent analysis.

### DNA and RNA Extraction

Total genomic DNA was extracted from the leaves using a Tiangen DNAsecure Novel Plant Genomic DNA Extraction Kit (Dp320-03) according to the manufacturer’s instructions (Tiangen, Beijing, China). Total RNA was extracted using an EASYspin Plus Polysaccharide Polyphenols/Complex Plant RNA Rapid Extraction Kit following the manufacturer’s instructions.

### Survey Analysis

Raw sequence data generated by the Illumina platform (San Diego, CA, United States) were filtered by the following criteria: filtered reads with adapter sequences, filtered reads with N bases >3, and filtered reads with low-quality bases (≤5) more than 20% (Li et al., 2009). The K-mer analysis was performed using jellyfish to estimate the genome size and sample heterozygosity. The genome size can be estimated using the K-mer analysis (Chikhi and Medvedev, 2014). The distribution of K-mer depends on the characteristic of the genome and follows Poisson distribution. We estimated the genome size of *S. dochna* using the following formula: genome size = (total number of 17-mer)/(position of peak depth).

### Genome Assembly and Quality Validation

#### Hi-Fi Assembly

We constructed a PCR-free SMRTbell library by repairing and connecting the high-quality purified genome and sequencing it by PacBio (Menlo Park, CA, United States) SMRT technology. After the library was constructed, its size was detected using an Agilent 2100 (Agilent Technologies, Santa Clara, CA, United States) fragment analyzer capillary electrophoresis or pulsed field electrophoresis. After the library was calculated by a PacBio calculator, sequencing primers and sequencing enzymes were combined into the SMRTbell template in proportion and then sequenced by diffusion loading. To obtain high-fidelity reads (Hi-Fi reads), we used SMRTlink software to conduct the subreads obtained previously for circular consensus sequencing (CCS) processing. The main parameters were min passes = 3 and min RQ = 0.99 (Sim et al., 2022).

The original data after sequencing were filtered and then assembled with hifiasm (Feng et al., 2021). First, an all vs. all comparison was used to correct the sequencing error. Second, after correction, a read overlap comparison was used again to construct a phased string graph. Finally, the contigs were generated according to the overlapping graph. The final genome sequence was obtained after de heterozygosity to generate de pseudo contigs (Koren et al., 2018).

#### Hi-C Assisted Genome Assembly

Raw image data files sequenced by a high-throughput sequencer (Illumina HiSeq 2500) were analyzed by base calling and transformed into sequenced reads. Raw sequencing data were stored in the FASTQ (Fq) file format. The raw reads obtained by sequencing contained a small number of articulated, repetitive, and low-quality reads, which could have affected the quality of comparison and the subsequent analysis. Therefore, we filtered the raw data to obtain clean reads. A total of 10,000 pairs of sequenced reads were randomly selected from the Hi-C sequencing library data and compared to the NT database using BLAST. The top 10 matched species in the output results were sorted and outputted to check for bacterial contamination. JUICER software was used to compare the Hi-C data with the sketched genome. Finally, the results of the Hi-C library were compared and analyzed using 3D DNA software (Durand et al., 2016). The scaffold number was obtained using these methods.

### Genome Annotation

For repeat element annotations, software RepeatMasker was used to mask the predicted repeats and known repeats (RepBase) in the genome. We used MITE Hunter, LTRharvest, LTR Finder, LTR retriever, and RepeatModeler to predict repeat sequences (Scott and Madden, 2004; Xiong et al., 2017).

We used reference protein sequences and RNA-Seq analysis to predict gene models. *Ab initio* gene prediction and annotation were performed by Augustus v3.318, SNAP, and GlimmerHMM. Augustus V3.0.3 combined with RNA-Seq data was used to predict the gene structure. First, parameters were trained with the training set. Intron hints were then obtained based on the comparison between RNA-Seq reads and the Scaffold (TopHat V2.0.10) (i.e., predicted intron location information) and then combined with intron hints for gene structure prediction. Second, SNAP and GlimmerHMM were used to predict the gene structure. The parameters were first trained with the training set, and then the genetic structure of the Scaffold shielded with repeated sequences was predicted (Ian, 2004). Third, Genemark-ET V4.57 combined with intron hints obtained from Augustus V3.0.3 was used to predict the genomic structure of the scaffold with repetitive sequences. The published protein sequences of *Oryza sativa*, *Zea mays*, *Echinochloa crus-galli*, *Brachypodium distachyon*, *S. bicolor*, and *Puccinellia tenuiflora* (NCBI) were used to perform homologous searches by GeMoMa-1.6.1.

For non-coding RNA prediction, we used tRNAscan-SE to predict the tRNA. rRNA and other types of ncRNA were searched with the Rfam database, and the specific information of ncRNA was obtained through similarity comparison.

For the gene functional annotation of protein-coding genes, we used six databases, including NR, Swiss-Prot, eggNOG, GO, KEGG, and InterPro, to perform function prediction. All these predictions of functions were integrated.

In this study, the corresponding gene function annotation results were obtained by comparing and analyzing a single database. Finally, a Venn analysis was performed by integrating the five databases to obtain the precise gene function annotation information.

### Comparative Genomic Analysis

#### Colinear Analysis and Phylogenetic Tree

MUMmer software can be used to quickly compare two genome sequences (Delcher et al., 2003). MUMmer was used to conduct genomic colinearity analysis on *S. dochna* and its related species *S. bicolor*. The parameter was “NucMER-G 1000-C90-L200.”

To identify the gene protein family, the OrthoMCL cluster analysis was adopted (Li et al., 2003). We performed all-VS-all BLAST alignments on protein-coding sequences of all the selected species (e-value was 1e^−5^ by default), calculated the similarity between sequences, and conducted a cluster analysis using the Markov clustering algorithm with an expansion coefficient of 1.5. The results of the protein family clustering were obtained. A Venn diagram was used to display the clustering results, which distinguished the endemic/common genes. The time standard point (correction point) was from the Timetree website.

Single-copy genes of each species were selected as reference markers for species with incomplete evolutionary studies, and quadruple degenerate sites were chosen to construct hypergenes. MAFFT software was used for multiple sequence comparisons of the hypergenes, and the most suitable base substitution model was selected. A phylogenetic tree was constructed based on the maximum likelihood method (ML) using RAxML software. Based on the single-copy gene family, McMctree (Burn-in = 5,000,000, sample-number = 1,000,000, and sample-frequency = 50) was used to estimate the differentiation time. The time standard point (correction point) was from the Timetree website (Hahn et al., 2005).

#### Gene Family Contraction and Expansion Analysis

Cafe software was used to analyze the gene families. This software can capture the changes in gene families between species based on random survival and death models combined with statistical inference methods. The number of contractions and expansions of gene families on each branch of evolution was obtained. We also determined whether contractions and expansions occurred in each gene family (Hahn et al., 2005; Hahn et al., 2007).

#### Positive Selected Gene Family Analysis

Early studies used the method of two-sequence alignment on all codons and the whole time zone before the divergence of the two sequences. The average value was utilized to calculate Ka and Ks. However, in fact, the vast majority of codons of a functional protein are in the process of evolution, and they are conservative most of the time. If a positive selection occurs, it will only affect some bits, and the positive selection only occurs during a specific time period. In 2002, a new method called the branch site model analysis method was reported, which can detect the positive events that occur in a specific evolutionary branch and affect only some positive selections (Jianzhi et al.). We used this method to detect the positive selection in protein coding sequences.

First, one-to-one orthology proteins from research species and related species were selected. Second, homologous protein sequences were compared with PRANK using the default parameters. Third, alignment results were filtered with G blocks with the following parameters: -t = c-e = . ft-b4 = 5-d = y. Fourth, CODEML in PAML was used to test the positive selection in a specific branch, which only affected some loci. Fifth, the Chi2 program in PAML was used to check and correct multiple hypotheses. Main parameters include degree of freedom = 2.

Based on these methods, we obtained the positive selection genes and proceeded with the GO enrichment analysis.

#### Whole-Genome Duplication

Whole-genome duplication (WGD) is typically associated with the rapid loss of repeated fragments, chromosome rearrangement, and the process of rearrangement back to the diploid. In this study, the distribution of synonymous substitutions (Ks) of each synonymous locus between adjacent homologous genes in the genome was constructed to detect WGD. We used BLASTP to compare the longest protein sequence of the gene in *S. dochna* genome and MCScanX to filter the comparison results. In addition, we used the yn00 tool in the PAML software package to calculate the synonymous replacement rate. The density distribution with the value of Ks was plotted for all paralog gene pairs. This approach is also known as the duplicate age distribution method (Vanneste et al., 2013). Synonymous mutations are generally considered neutral and gradually accumulate in the genome at a nearly constant rate. Therefore, Ks can represent collateral homologous genes.

## Results

### High-Quality Gene Assembly

The quality control results of the offline data revealed 43.7 Gb of clean bases with a GC content of 43.52% and 146,092,905 clean reads (Supplementary Table S9). A K-mer analysis revealed that *S. dochna* is a heterozygous species (0.619%), and the 17-mer frequency distribution plot is shown as Supplementary Figure S1.

PacBio long reads (Nurk et al., 2020) and Illumina short reads (Dudchenko et al., 2017; Vurture et al., 2017) technologies were used to assemble the *S. dochna* genome. The PacBio clean subread statistical results are shown in Supplementary Table S7. We assembled the genome sequences into 1,628 contigs with a total length of 831.09 Mb, a contig N50 length of 533.94 kb, and the longest contig of 822.40 kb after the initial assembly (Supplementary Table S1). Thus, contigs with an average GC content of 55–57% (abnormal GC content peak in the figure) were processed by filtering the contigs. We obtained the final genome as 144 contigs with a total length of 777.99 Mb, a contig N50 of 553.47 kb, and the longest contig of 822.40 kb, which is 47 Mb bigger than that of *S. bicolor* (∼730 Mb), suggesting a close relationship between *S. dochna* and *S. bicolor*. The results of the Hi-C library were analyzed using 3D DNA software, and the results revealed a genome that was 778.03 Mb long with scaffold N50 of 727.11 kb. The third-generation assembly results are shown in Table 1. The evaluation results of the Benchmarking Universal Single-Copy Ortholog (BUSCO) analysis indicated 97.5% completeness. A complete Single-Copy BUSCO further validated the high degree of completeness of the *S. dochna* genome assembly (Supplementary Table S2).

**TABLE 1:** Genome assembly results.

Parameter	Contig	Scaffold
Genome assembly and Hi-C results	144	82
Total number	777,990,620	778,026,804
Total length (bp)	55,347,497	43,657,906
N50 length (bp)	43.90	43.90
GC (%)	11,660,912	—
Contig N90 length (bp)	—	72,771,365
Scaffold N50 length (bp)	—	94.09
Chromosome length (%)	—	—

Hi-C, high-throughput chromosome capture technology.

The length distribution statistics of Hi-Fi reads and Hi-Fi read bases are shown in Supplementary Figures S4A,B. The length of most Hi-Fi reads was distributed between 1,000 and 2,000. The gene assembly results demonstrate the high quality of the *S. dochna* genome assembly.

The completeness and accuracy of the assembly quality were assessed using the sequence data return ratio, GC-depth evaluation, and BUSCO evaluation. First of all, the results of our second-generation return ratio showed a mapping ratio of 99.58%, suggesting that most of the *S. dochna* genome had been assembled (Table 2). Second, an evaluation of the depth of GC found that there were no separate scattered clusters on the figure, which proved that our assembly results were not polluted. A BUSCO evaluation was used to evaluate the completeness of the *S. dochna* genome (Waterhouse et al., 2018).

**TABLE 2:** Statistics of the results of a comparison of the DNA library.

Sample name	Reads number	Mapped	Properly paired Mapped	Mapped DifferentChr	Mapped different ChrMapQ>=5	Secondary reads
*Sorghum dochna*	294,034,737	292,796,203	279,197,414	9,547,568	4,598,204	1,848,927
		99.58%	95.55%	3.2%	1.6%	0.6%

**FIGURE 2:**
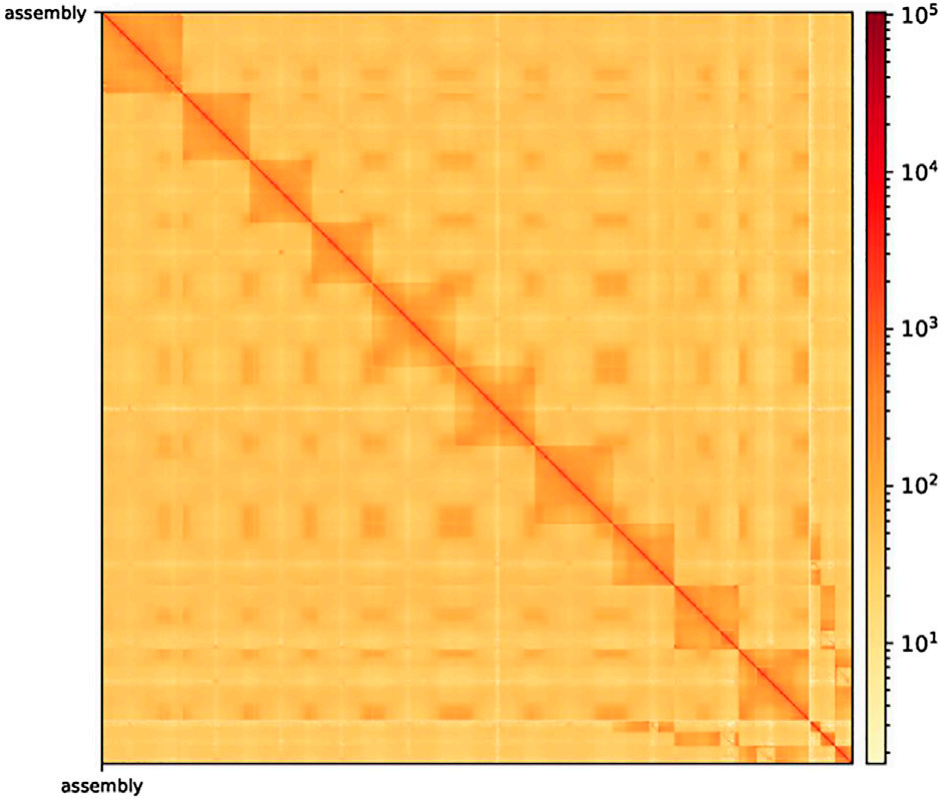
Hi-C-assisted assembly genome interaction heatmap. The exchange within chromosomes is stronger than that between chromosomes. Moreover, the exchange between the same chromosomes with a close physical location is stronger than the exchange between chromosomes with a distant physical location. Hi-C, high-throughput chromosome capture technique.

After Hi-C assembly, 10 chromosomes were assembled, and 751 Mb genomes were fixed to further verify the accuracy of the assembly results. This included 94.09% gene content and involved calculating the exchange between and within chromosomes. The heatmap in Figure 2 shows the intergenomic exchange (Zhang et al., 2013).

### Genome Annotation

#### Repeat Sequence Statistics

The results of annotation showed that Class Ⅰ retrotransposons accounted for the highest proportion of the repeated sequences. The long terminal repeats (LTRs) were the most abundant transposable elements (TEs). LTR-retrotransposons accounted for 59.22%, and Gypsy accounted for 47.46% in the LTR–retrotransposons. In contrast, Copia accounted for 6.79%. Notably, Gypsy-type and Copia-type TEs accounted for most of the LTRs (Supplementary Table S6). Non-LTR-retrotransposons accounted for 5.97%, whereas Class II DNA transposons accounted for 9.3% of the repeated sequences (Supplementary Table S4).

### Coding Gene and Non-Coding RNA Predictions

We predicted that 37,971 genes were encoded, and there were 39,937 transcripts in *S. dochna*. In addition, the number of genes in *S. dochna* was higher than the number of genes in *O. sativa*, *Z. mays*, *E. crus-galli*, *B. distachyon*, *S. bicolor*, and *P. tenuiflora* (Supplementary Table S3), which was similar to that annotated for *S. bicolor*, indicating that its genome is more complex. Based on the open reading frames, we predicted 20,108 genes in *S. dochna* (Supplementary Table S5). Moreover, according to the types of ncRNAs, the results of the ncRNA classes are shown in Supplementary Table S6. There were three sRNAs, 3,101 rRNAs, 172 miRNAs, and 847 tRNAs. There were 5,694 snRNA:: snoRNA:: CD-Box in the ncRNA.

### Gene Functional Annotation

**FIGURE 3:**
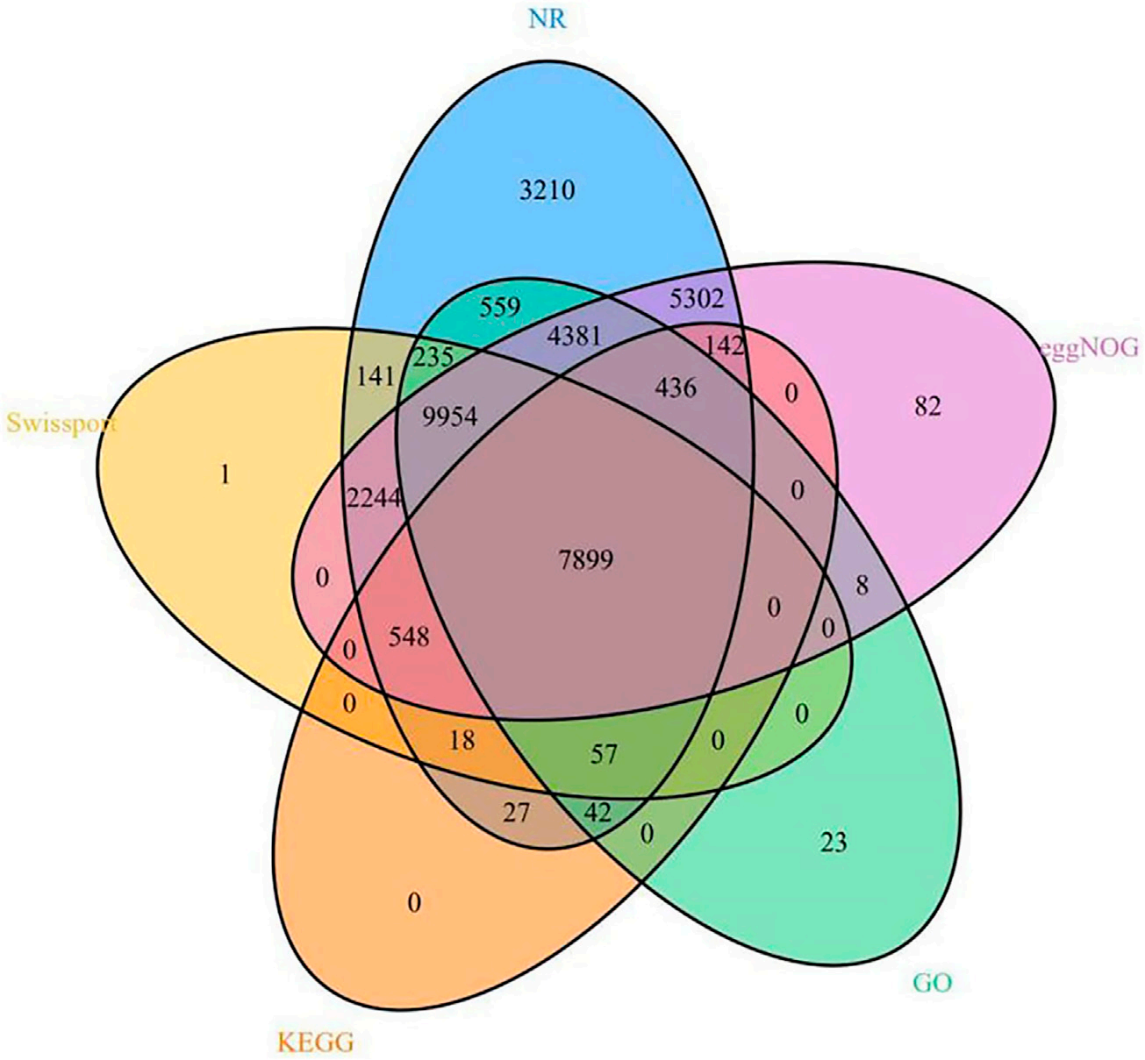
A Venn analysis of gene function annotation.

We annotated 35,309 types of gene information using six databases (NR, Swiss-Prot, eggNOG, GO, KEGG, and InterPro). The corresponding gene function annotation results were obtained by comparing the analyses of a single database. A total of 35,195 types of gene information were annotated by NR (Supplementary Table S13), and 21,097 types of gene information were annotated by Swiss-Prot (Supplementary Table S14). A total of 9,169 types of gene information were annotated by KEGG (Supplementary Table S12), and 23,594 types of gene information were annotated by GO (Supplementary Table S11). A total of 30,996 types of gene information were annotated by eggNOG (Supplementary Table S10). Finally, a Venn analysis was conducted by integrating the five databases (NR, Swiss-Prot, eggNOG, GO, and KEGG), which revealed a set of 7,988 common gene annotations (Figure 2 and Supplementary Table S15). Venn analysis of gene functional annotations was shown in Figure 3.

### Genome Comparison

#### Colinearity and Phylogenetic Relationships

**FIGURE 4:**
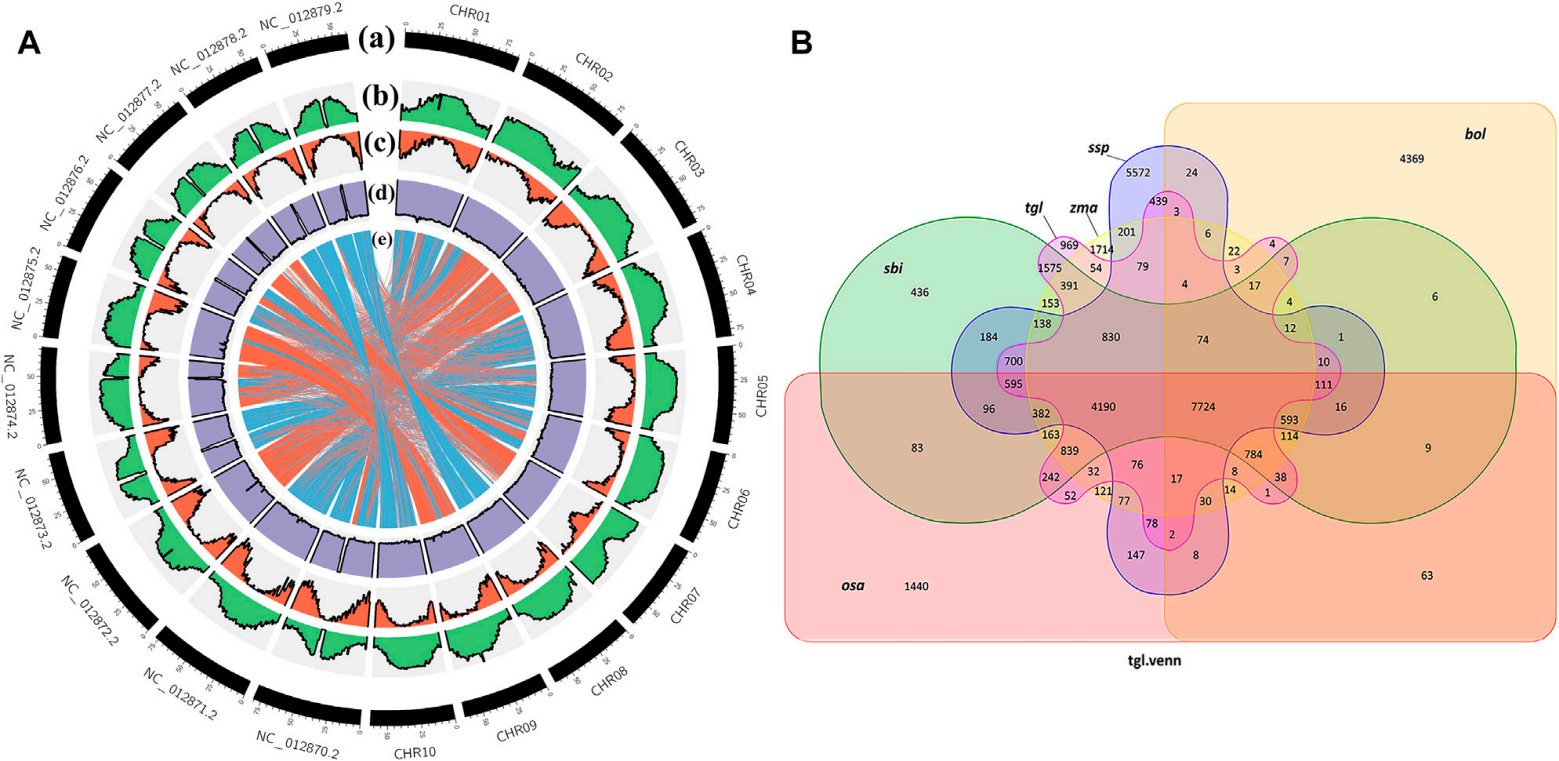
**(A)** Circos display of the important features of the assembled *Sorghum dochna* genome. From outside to inside, **(A)** chromosome, **(B)** repeat sequence distribution, **(C)** gene distribution, **(D)** GC content distribution, and **(E)** colinearity between *S. dochna* and *S. bicolor*. **(B)** Venn diagram of the protein families. tgl: *S. dochna* (*S. bicolor dochna*), sbi: *S. bicolor* (*S. bicolor bicolor*), osa: rice (*Oryza sativa*), zma: maize (*Zea mays)*, ssp: sugarcane (*Saccharum spontaneum*), and bol: kale (*Brassica oleracea*).

When species are closely related, there is greater coverage of colinear segments on the genome, and the colinear relationship between the genomes of different species is more accurate (Krzywinski et al., 2009). Figure 4A shows that the colinear relationship between *S. dochna* and *S. bicolor* is relatively strong, and their relationship is relatively close. Circos displays the important features of the assembled *S. dochna* genome.

#### Gene Protein Family Identification and Positive Selection Gene Analysis

A protein family is a group of proteins with certain similarities in sequence and function. A protein family clustering analysis (of predicted gene proteins) groups proteins with identical or similar functions together, thus reducing the complexity of further analyses. The comparison with exogenous organisms also helps to understand and predict the gene functions. In the current genome of the Gramineae members *S. bicolor*, *S. officinarum*, *Z. mays*, and *O. sativa*, which are closely related to *S. dochna*, since they have substantial continuity in genome assembly continuity, they are selected for the protein family analysis. Among them, *S. officinarum* and *S. dochna* have the same biological characteristics of high sugar content. Simultaneously, distant species *Brassica oleracea* was selected for comparison. Figure 4B shows the Venn diagram of protein clustering in *S. dochna* and other species. Among them, 969 gene families were specific to *S. dochna*. A total of 1,440 were specific to *O. sativa*, 1,714 were specific to *Z. mays*, 436 were specific to *S. bicolor*, 5,572 were specific to *S. officinarum*, and 4,369 were specific to *B. oleracea*. Distributions of the numbers of single-copy genes, multi-copy genes, endemic genes, and other types of genes per species are shown in Supplementary Figure S2.

**FIGURE 5:**
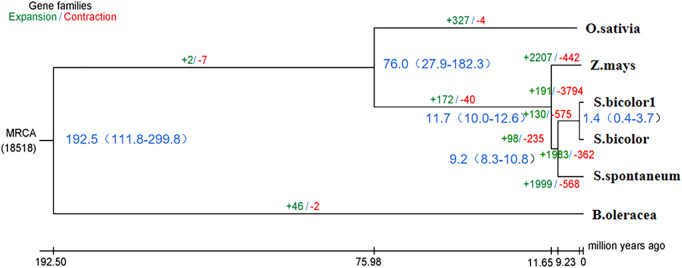
Phylogenetic tree of the species. In the analysis that estimated the time of differentiation of species, the branch length obtained is the base replacement rate, and after the analysis of species differentiation time, the branch length is the time in million years. *O. sativa*: *Oryza sativa*. *Z. mays*: *Zea mays*. *S. bicolor1*: *Sorghum dochna*. *S. bicolor*: *Sorghum bicolor. S. spontaneum*: *Saccharum spontaneum. B. oleracea*: *Brassica oleracea.*

Phylogenetic trees that were constructed based on protein clustering results showed that *S. dochna* was closer to *S. bicolor*, while it was the most distant from *B. oleracea* (Figure 5). Based on the differentiation time of species, *S. bicolor* and *S. dochna* diverged from sugarcane (*Saccharum officinarum*) 9.2 and 1.4 million years ago, respectively. A cafe software analysis showed that 191 gene families were significantly expanded, while 3,794 were significantly contracted in *S. dochna* (tgl) after the family-wide *p*-value threshold was 0.05. The result of GO enrichment in expanded gene families is shown in Supplementary Figure S3. A GO function enrichment analysis of these gene families revealed that the expanded gene families were primarily clustered in the metabolic process, DNA reconstruction, and DNA binding among others (Supplementary Figure S5). The positive selection analysis model with *S. dochna* as the foreground branch and other species as the background branch was established. Finally, we obtained four significant positive selected genes. The GO enrichment analysis showed that these positive selection genes were primarily clustered in organic cyclic compound binding, nucleic acid binding, nucleotidyl transferase activity, and tRNA methylation among others (Supplementary Figures S5, S6). One significant positive selection gene was clustered in peptidyl-prolyl *cis-trans* isomerase (PPIase) activity. The GO enrichment information is shown in Supplementary Table S8.

According to the species differentiation time, *S. dochna* and *S. bicolor* diverged 1.4 million years ago. During this period, the temperature of the Earth was lower by 5–10°. Since then, the Earth has undergone several alterations in climate. *S. bicolor* is native to Africa, while *S. dochna* is native to India/Myanmar, which is currently separated by the Indian Ocean (Dutt, 1999). Therefore, it is hypothesized that the formation and differentiation of the two *S. bicolor* species could be related to the climate and tectonic plate movement at that time (Chase, 1978). However, we did not explore the similarities in physiological functions and gene family clustering between the two *S. bicolor* species in more detail. Therefore, further studies should be conducted to fully elucidate their specific biological properties (Hahn et al., 2005).

During the GO enrichment analysis, one significant positive selection gene was clustered in peptidyl-prolyl *cis-trans* isomerase (PPIase) activity (Supplementary Figure S6). PPIase can catalyze the conformation of protein substrates or the N-terminal of proline residues in the polypeptide from a homeopathic structure to a trans structure (Maruyama et al., 2000). This type of protein can also improve the stress resistance of plants when they are in adversity and pass on the stress resistance to future generations. Therefore, it is hypothesized that the high stress resistance of *S. dochna* is related to the positive selection of this gene. Other positive selection genes were clustered in tRNA methylation. tRNA methylation primarily occurs in the nitrogen atom of tRNA and can also occur in the oxygen atom of the 2′ hydroxyl of nucleotide ribose ring (Gustilo et al., 2008). In addition, the 5′ carbon atom on purine and the 2′ and 8′ carbon atoms on adenosine have also been identified. The methylation phenomenon is primarily related to protein translation and the stability of tRNA (Motorin and Helm, 2011). In addition, for organic cyclic compound binding, *S. dochna* is a high-quality bio-energy crop with high sugar content. Most sugar structures are constituted with organic cyclic compounds, such as furan and pyran. Therefore, we hypothesized that during the evolution of sweet sorghum, positive selection genes were enriched in the binding of organic cyclic compounds, which could be used in the synthesis of sugars (Lingle et al., 2012).

#### Whole-Genome Duplication Analysis

Whole-genome duplication (WGD) is often associated with a rapid loss of repeated fragments and chromosome rearrangements. Notably, it provides new materials for the evolution of organisms, particularly plants, which assists them in their adaptation to new environments. A whole-genome duplication analysis performed on the pan-genome of *S. dochna* (Figure 5) revealed gene replication and loss and a sudden increase in the Ks within a certain period (shown as a peak), suggesting that a WGD event could have occurred. Otherwise, loss occurred (shown as a smooth decline).

**FIGURE 6:**
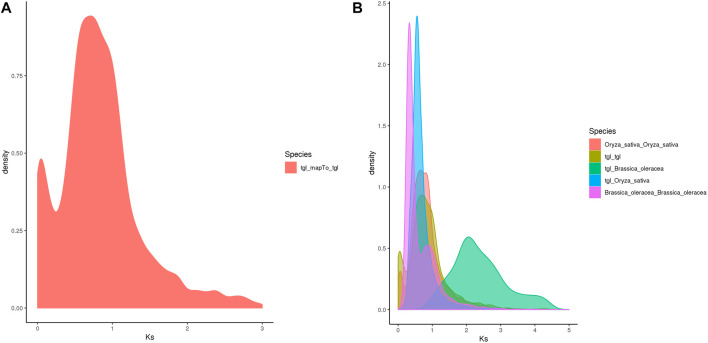
**(A)** Ks distribution map of *Oryza sativa*. The peaks indicate that WGD events occurred during the evolution of species. **(B)** Ks distribution map of the complete genome replication of *Sorghum dochna* and its related species. Tgl: *S. dochna*. WGD, whole-genome duplication.

The Ks of ortholog gene pairs between the *S. dochna* genome and those of related species were searched for a density distribution map. The Ks distribution of orthologs (Figures 6A,B) suggested that a WGD event occurred in *S. dochna* as in other species of the Gramineae family. As shown in Figure 5A, two differentiation events occurred in *S. dochna* when the Ks values were 0.1 and 0.8. Simultaneously, Figure 5B shows that the green one represents the differentiation event of *S. dochna* and *B. oleracea*, and *S. dochna* and *B. oleracea* had the highest Ks values. Therefore, the WGD event occurred the earliest in these two species, followed by *S. dochna* and *O. sativa*, while the differentiation of *O. sativa*, *B. oleracea*, and *S. dochna* occurred relatively late. Thus, the Ks value was relatively low.

## Conclusion

In this study, we used PacBio long reads, Illumina short reads, and Hi-C sequences to assemble the *S. dochna* genome and analyze its gene family and relationship with other species. Our findings provide a preliminary understanding of the *S. dochna* genome. A high-quality chromosome assembly was achieved using PacBio long reads, Illumina short reads, and Hi-C sequences. The genome size of *S. dochna* is 777 Mb, with a contig N50 of 553.5 kb and a GC content of 43.9%. The coding gene analysis revealed 37,971 genes and 39,937 transcripts in the *S. dochna* genome.

The genome comparison indicated that *S. dochna* and *S. bicolor* had the strongest colinearity. GO enrichment revealed that the positive selection genes primarily clustered in organic cyclic compound binding, nucleic acid binding, nucleotide transferase activity, and tRNA methylation among others. However, the synthetic pathway of sugar production in *S. dochna* is still unclear (Hakim and Wijaya, 2009). Thus, subsequent studies on genome exploration should focus on the transcriptome and proteome of *S. dochna*. In addition, only one variety of *S. dochna* was used in this study. Cognizant of this, future studies should use multiple varieties to comparatively analyze the species and construct reference-quality genome sequences.

## Data Availability

The datasets presented in this study can be found in online repositories. The names of the repository/repositories and accession number(s) can be found in the article/Supplementary Material.
