# Supplementary figures and images for: A High-Quality Genome Assembly of Sorghum dochna
Source: Front Genet. 2022 Aug 12;13:844385. doi: 10.3389/fgene.2022.844385 (PMC9412107; doi:10.3389/fgene.2022.844385)

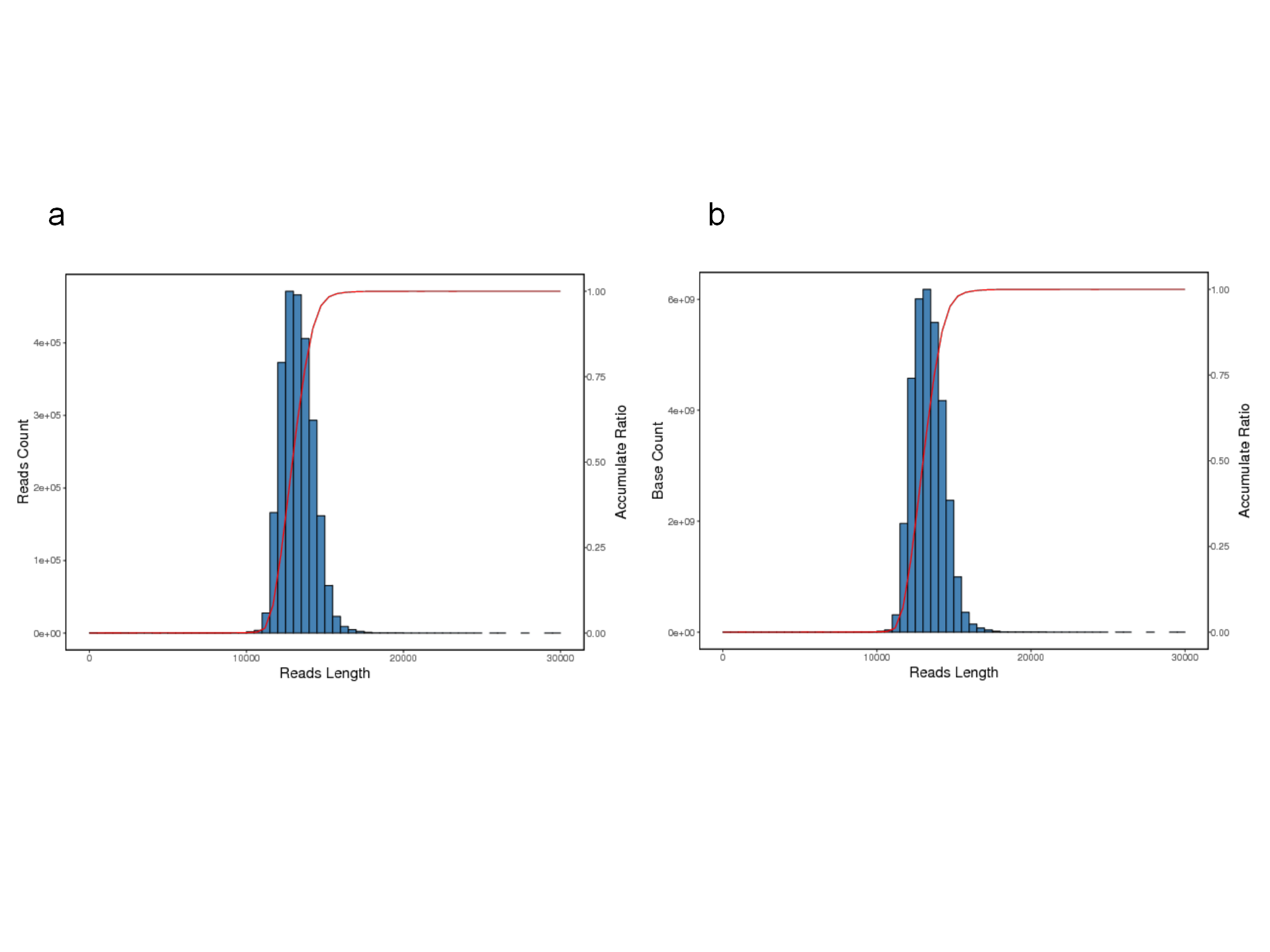

Supplement: Supplementary file 1 [file Image4.JPEG]

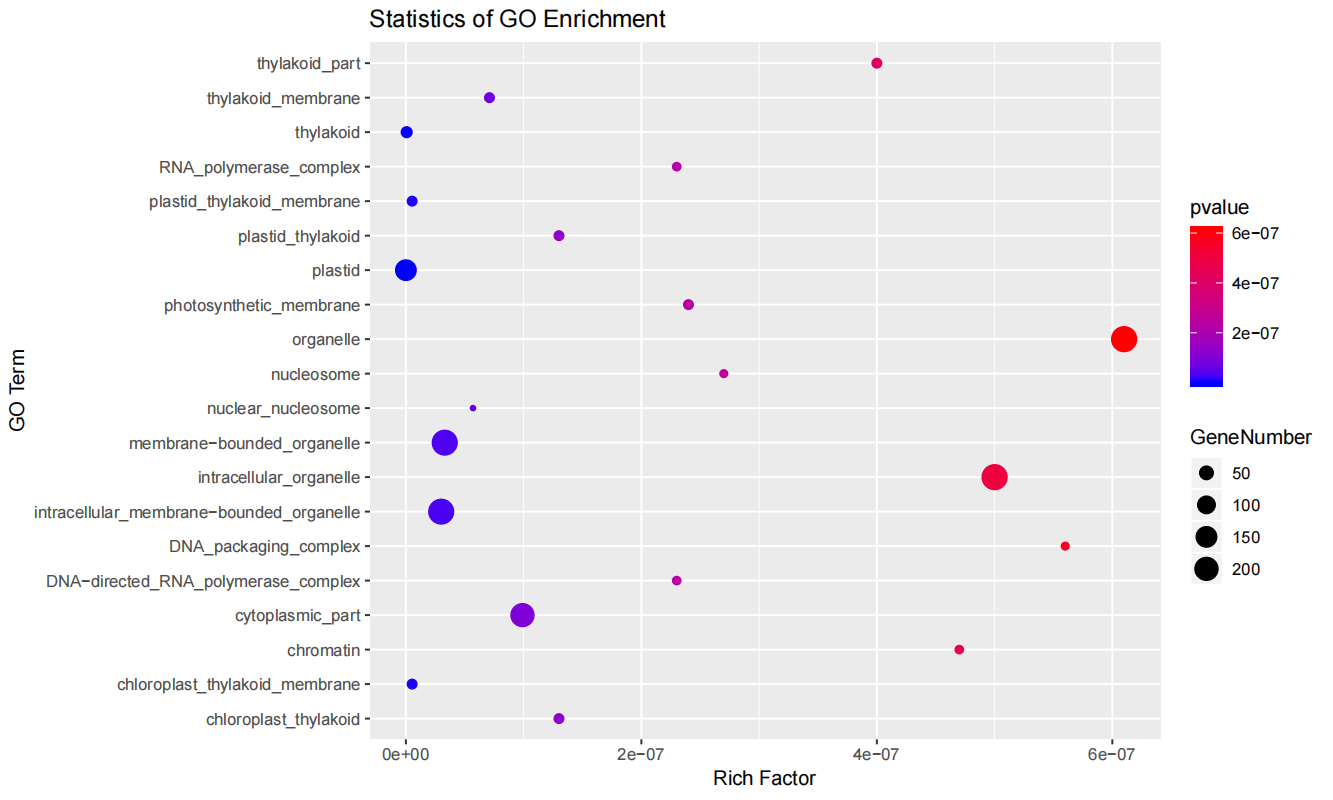

Supplement: Supplementary file 2 [file Image5.JPEG]

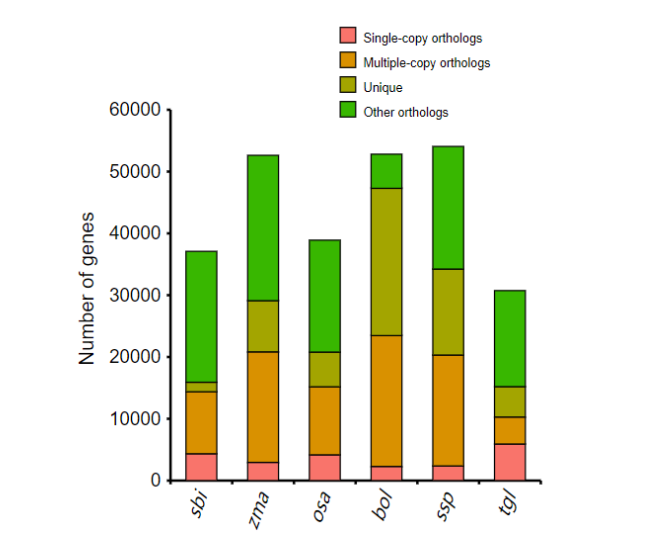

Supplement: Supplementary file 3 [file Image2.PNG]

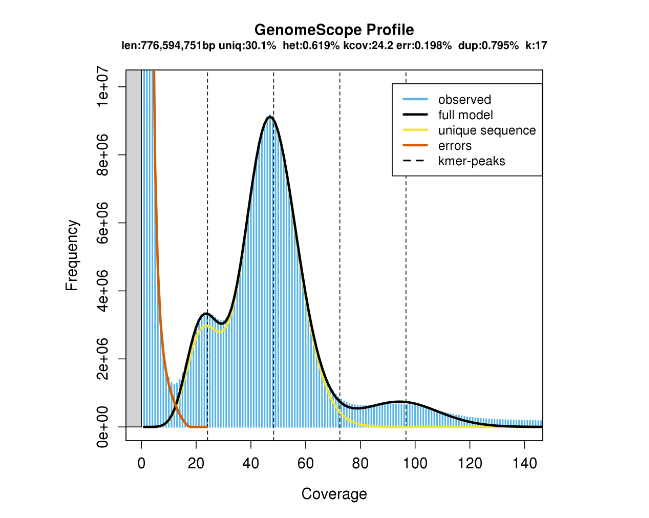

Supplement: Supplementary file 5 [file Image1.PNG]

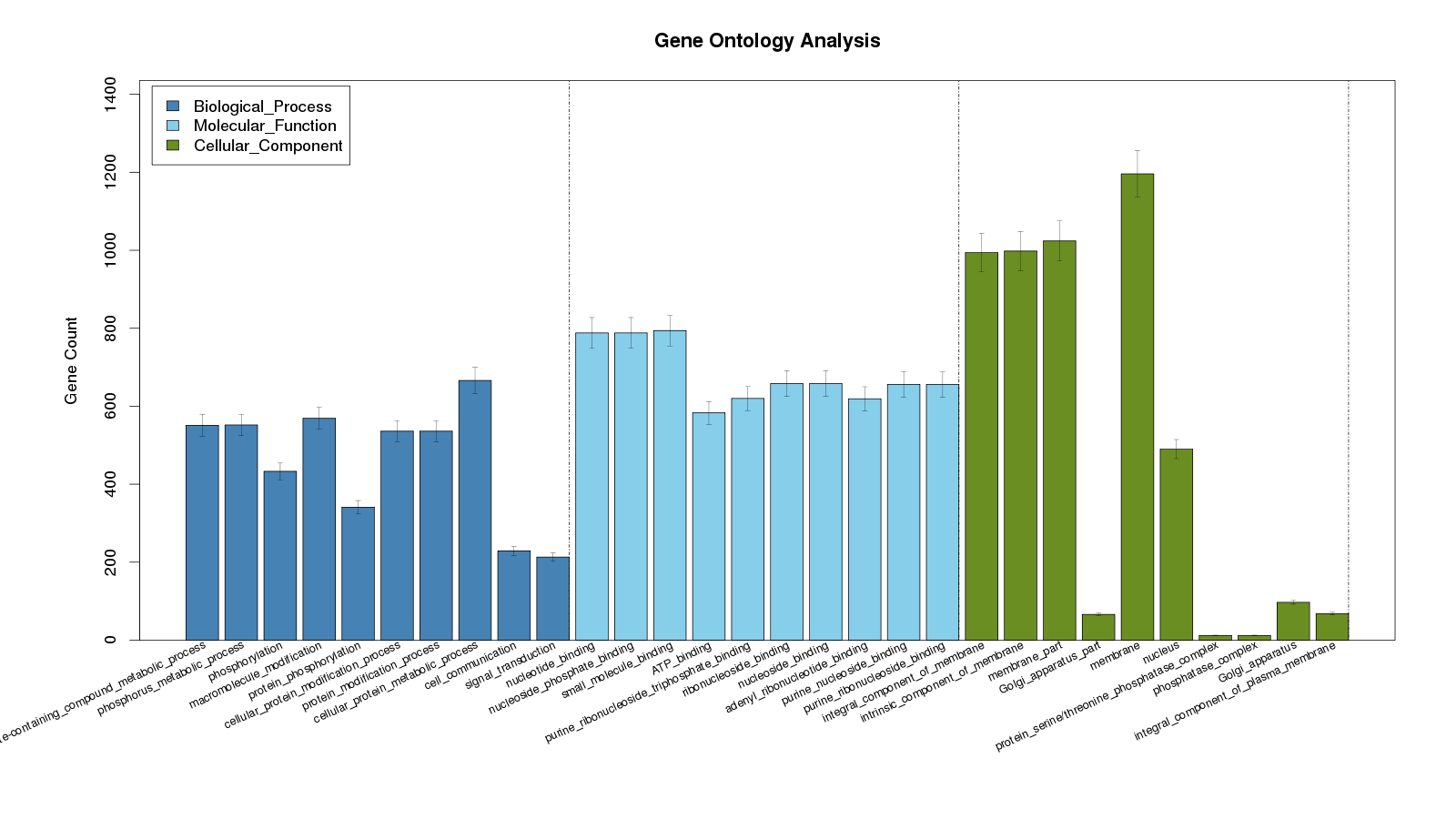

Supplement: Supplementary file 6 [file Image3.PNG]

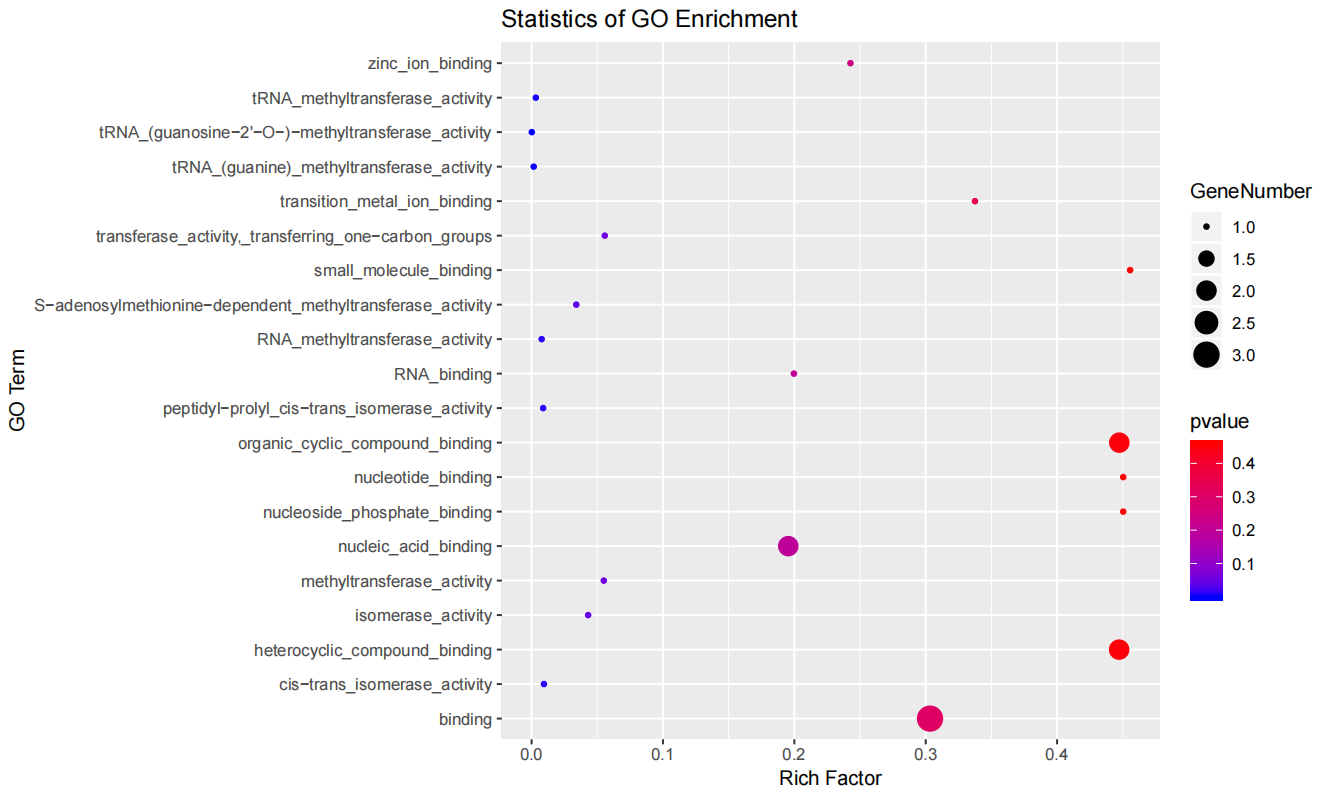

Supplement: Supplementary file 7 [file Image6.JPEG]
